# Water-Soluble Visible Light Sensitive Photoinitiating System Based on Charge Transfer Complexes for the 3D Printing of Hydrogels

**DOI:** 10.3390/polym13183195

**Published:** 2021-09-21

**Authors:** Hong Chen, Mehdi Vahdati, Pu Xiao, Frédéric Dumur, Jacques Lalevée

**Affiliations:** 1 Université de Haute-Alsace, CNRS, IS2M UMR 7361, F-68100 Mulhouse, France; hong.chen@uha.fr (H.C.); mehdi.vahdati@uha.fr (M.V.); 2 Université de Strasbourg, F-67200 Strasbourg, France; 3 Research School of Chemistry, Australian National University, Canberra, ACT 2601, Australia; 4 Aix Marseille Univ, CNRS, ICR UMR 7273, F-13397 Marseille, France

**Keywords:** charge transfer complexes, photopolymerization, water-soluble photoinitiating system, visible light

## Abstract

The development of visible-light 3D printing technology by using water-soluble initiating systems has attracted widespread attention due to their potential applications in the manufacture of hydrogels. Besides, at present, the preparation of water-soluble photoinitiators suitable for visible light irradiation (such as LEDs) still remains a challenge. Therefore, this work is devoted to developing water-soluble photoinitiators (PI)/photoinitiating systems (PIS) upon irradiation with a LED @ 405 nm. In detail, a new water-slightly-soluble chalcone derivative dye [(*E*)-3-(4-(dimethylamino) phenyl)-1-(4-(2-(2-(2-methoxyethoxy) ethoxy) ethoxy) phenyl) prop-2-en-1-one] was synthesized here and used as a PI with a water-soluble coinitiator, i.e., triethanolamine (TEA) which was also used as an electron donor. When combined together, a charge transfer complex (CTC) formed immediately which exhibited excellent initiating ability for the free radical photopolymerization of poly(ethyleneglycol)diacrylate (PEG-DA). In light of the powerful CTC effect, the [dye-TEA] CTC could not only exhibit enhanced water solubility and mechanical properties but could also be effectively applied for 3D printing. This CTC system is environmentally friendly and cost-saving which demonstrates a great potential to prepare hydrogels via photopolymerization.

## 1. Introduction

During the last decades, photopolymerization has been the focus of intense research efforts due to the wide range of applications in which this polymerization technique is involved. Photopolymerization exhibits several appealing features, such as the possibility to carry out the polymerization reaction at room temperature, without any organic solvent. The polymerization process can also be extremely fast, enabling it to reach high fabrication speed and making photopolymerization suitable for biomedical applications (e.g., tissue engineering, drug delivery systems, medical devices, etc.) [1,2,3]. The combination of this process with 3D printing technology can provide an efficient tool to elaborate structures with a high precision of shape using hydrogels and enabling to achieve the fusion of structure and function. As the photoinitiator/photoinitiating system (PI/PIS) is the key element for performing highly efficient photopolymerization, the development of new PI/PIS is still a challenge [4]. In addition, one of the basic requirements of photopolymerization systems for biomedical applications is to use water-soluble PI/PIS as the water is environmentally friendly, non-toxic, and an inexpensive solvent that can overcome many of the problems associated with classic organic compositions [5,6]. Therefore, the design and synthesis of PI or PIS with good water solubility, non-toxicity, high photoactivity, high initiation efficiency, low volatility and high migration stability is still a research hotspot in this field.

2-Hydroxy-4′-(2-hydroxyethoxy)-2-methylpropiophenone (Irgacure 2959) is the most commonly reported water-soluble PI and this photocleavable molecule is extensively used for the preparation of hydrogels. However, its limited water-solubility (≤0.5 wt%) and its UV light sensitivity are clearly undesirable for some biomedical applications involving living cells. Parallel to this, this PI is also inapplicable for deep curing under visible light irradiation [7,8]. In order to obtain three-dimensional hydrogels suitable for biomedical applications through 3D printing technology, a visible light source and water-soluble PI/PIS are required.

**Scheme 1:**
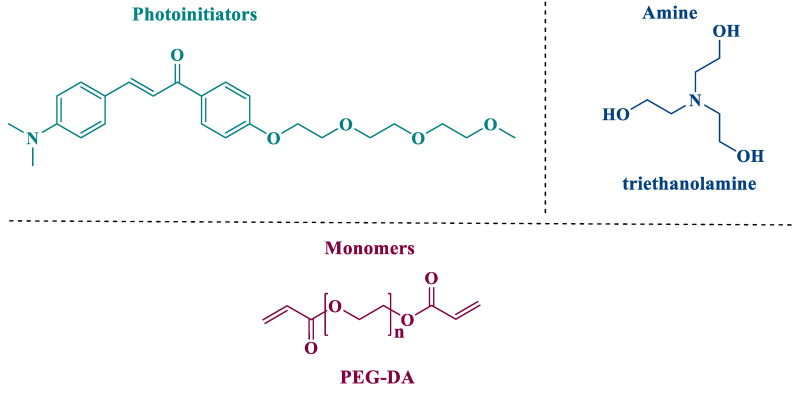
Chemical structures of the chalcone based-dye, triethanolamine (TEA) and the functional benchmark monomer (PEG-DA) used in this work.

Recently, in parallel to the traditional approaches consisting of developing water-soluble photoinitiating systems by means of chemical engineering and often at the origin of molecules designed by mean of complex synthetic routes [9,10], a simpler, more effective and elegant method based on converting water-insoluble PI into visible light water-soluble PI by the formation of a charge-transfer complex (CTC) has been proposed [11]. The resulting CTCs can show good water solubility and high efficiency under visible light irradiation. Charge transfer complexes sometimes named electron donor−acceptor (EDA) complexes, are the result of a ground state (noncovalent) association between an electron donor and an electron acceptor, giving rise to an intermolecular electronic charge-transfer transition and inducing a red-shift of the light absorption properties compared to that of the parent structures [12]. CTCs have notably been reported in the literature as visible PIS for radical and cationic photopolymerization [11,12,13,14,15]. In the present work, we propose to explore a novel CTC based on a slightly water-soluble chalcone-based natural dye (used as an electron acceptor) and the water-soluble triethanolamine (TEA) (used as an electron donor). This CTC was notably used as a water-soluble photoinitiating system (PIS) under visible light (see Scheme 1). Such a simple approach consisting in mixing dye and TEA in order to generate water-soluble photoinitiating systems was determined as being an effective, safe, and environmentally friendly approach for elaborating PIS capable to initiate free radical polymerization. In detail, the water solubility and the photopolymerization efficiency of the CTC complex were studied. Finally, 3D direct laser write experiments were carried out with the proposed dye/TEA PIS, and excellent 3D patterns could be obtained.

## 2. Materials and Methods

### 2.1. Chemical Compounds

The free radical photopolymerization monomers used in this work, namely, polyethylene glycol diacrylate (PEG-diacrylate: SR 610) was purchased from Sartomer-Europe (Colombes, France). The water-soluble coinitiator triethanolamine (TEA) (used as an electron donor) was purchased from Sigma-Aldrich (Saint-Quentin-Fallavier, France). Their corresponding molecular structure were shown in Scheme 1. The solvent (i.e., acetonitrile) was also purchased from Sigma-Aldrich and was of analytical grade. The slightly water-soluble photoinitiator (dye) was prepared as described below and its chemical structure is shown in Scheme 1.

#### 2.1.1. Synthesis of the Water Soluble Chalcone

Details concerning the solvents, chemicals, NMR acquisitions and the control of purity are given in the reference [16].

#### 2.1.2. Synthesis of 2-(2-(2-Methoxyethoxy)ethoxy)ethyl Methanesulfonate

**Scheme 2:**
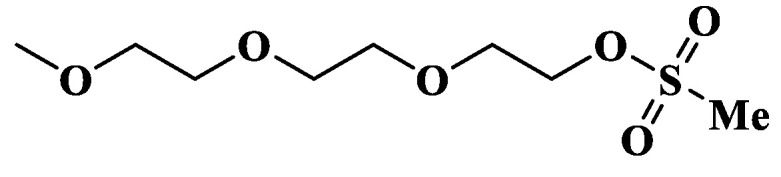
2-(2-(2-methoxyethoxy)ethoxy)ethyl Methanesulfonate.

Triethyleneglycol monomethyl ether (5 mL, 31 mmol) and triethylamine (7 mL) were dissolved in dry chloroform (70 mL). Methanesulfonyl chloride (4.58 g, 3.01 mL, 40 mol, M = 114.55 g/mol, d = 1.48) was added under N_2_. The solution was stirred for 24 h at room temperature. The organic layer was washed with distilled water, dried over magnesium sulfate and the solvent removed under reduced pressure. It was used without any further purification in the next step (7.13 g, 95% yield). ^1^H NMR (CDCl_3_) δ: 4.40–4.34 (m, 2H), 3.80–3.71 (m, 2H), 3.69–3.57 (m, 6H), 3.54–3.49 (m, 2H), 3.37 (d, *J* = 3.3 Hz, 3H), 3.06 (d, *J* = 3.9 Hz, 3H); ^13^C NMR (CDCl_3_) δ: 71.91, 70.64, 70.54, 70.53, 69.28, 69.02, 59.01, 37.71. Analyses were consistent with those previously reported in the literature [17] (See Scheme 2).

#### 2.1.3. Synthesis of 1-(4-(2-(2-(2-Methoxyethoxy)ethoxy)ethoxy)phenyl)ethan-1-one

**Scheme 3:**
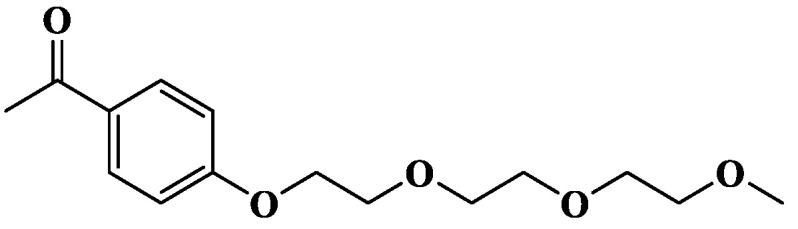
1-(4-(2-(2-(2-methoxyethoxy)ethoxy)ethoxy)phenyl)ethan-1-one.

4′-Hydroxyacetophenone (5 g, 36.72 mmol, M = 136.15 g/mol) was dissolved in dry THF (100 mL) and sodium hydride 60% (2.2 g, 1.5 eq. M = 24 g/mol) and the solution was stirred to room temperature for 5 min. Then, 2-(2-(2-methoxyethoxy)ethoxy)ethyl methanesulfonate (9.8 g, 1.1 eq., M = 242.29 g/mol) was added and the solution was stirred at room temperature overnight. The solution was quenched with water and the solvent was removed under reduced pressure. The residue was dissolved in dichloromethane. The organic phase was washed with water, dried over magnesium sulfate and the solvent removed under reduced pressure. The residue was filtered on a plug of silica gel using dichloromethane as the eluent (9.12 g, 88% yield). ^1^H NMR (CDCl_3_) δ: 7.96–7.86 (m, 2H), 6.96–6.84 (m, 2H), 4.19 (td, *J* = 4.8, 1.0 Hz, 2H), 3.88 (td, *J* = 4.8, 1.1 Hz, 2H), 3.74 (ddd, *J* = 6.3, 3.5, 1.0 Hz, 2H), 3.70–3.62 (m, 4H), 3.57–3.52 (m, 2H), 3.40–3.35 (m, 3H), 2.58–2.52 (m, 3H); ^13^C NMR (CDCl_3_) δ: 196.77, 162.73, 130.55, 114.27, 71.93, 70.90, 70.66, 70.59, 69.54, 67.62, 59.04, 26.33. Analyses were consistent with those previously reported in the literature [17] (See Scheme 3).

#### 2.1.4. Synthesis of 3-(4-(Dimethylamino)phenyl)-1-(4-(2-(2-(2-methoxyethoxy)ethoxy)ethoxy) phenyl)prop-2-en-1-one

**Scheme 4:**
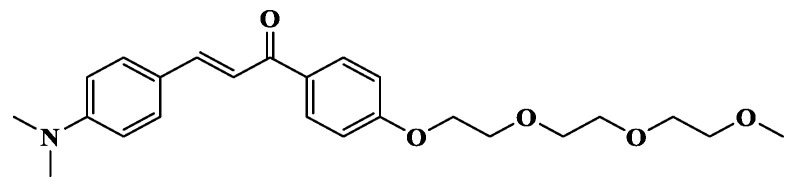
3-(4-(dimethylamino)phenyl)-1-(4-(2-(2-(2-methoxyethoxy)ethoxy)ethoxy) phenyl)prop-2-en-1-one.

4-Dimethylaminobenzaldehyde (2.98 g, 20 mmol, M = 149.19 g/mol) and 1-(4-(2-(2-(2-methoxyethoxy)ethoxy)ethoxy)phenyl)ethan-1-one (5.65 g, 20 mmol, M = 282.34 g/mol) were dissolved in ethanol (200 mL). The solution was cooled into ice and aq. KOH 40% (20 mL) was added. The solution was stirred at room temperature overnight. HCl (6N) was added until pH = 7 and the solvent was removed under reduced pressure. The residue was extracted with dichloromethane. The organic phase was washed with water several times. The organic phase was dried over magnesium sulfate, and the solvent removed under reduced pressure. The residue was purified by column chromatography (SiO_2_) using a gradient of solvent from dichloromethane to acetone (5.87 g, 71% yield). ^1^H NMR (CDCl_3_) δ: 8.01 (d, *J* = 8.9 Hz, 2H), 7.78 (d, *J* = 15.4 Hz, 1H), 7.54 (d, *J* = 8.9 Hz, 2H), 7.34 (d, *J* = 15.4 Hz, 1H), 6.98 (d, *J* = 8.9 Hz, 2H), 6.69 (d, *J* = 8.9 Hz, 2H), 4.20 (dd, *J* = 9.8, 4.9 Hz, 2H), 3.92–3.86 (m, 2H), 3.77–3.62 (m, 6H), 3.58–3.52 (m, 2H), 3.38 (s, *J* = 1.7 Hz, 3H), 3.04 (s, 6H); ^13^C NMR (CDCl_3_) δ: 188.91, 162.20, 151.91, 144.94, 132.00, 130.55, 130.50, 130.26, 122.92, 116.74, 114.28, 111.87, 71.94, 70.90, 70.67, 70.59, 69.60, 69.54, 67.59, 59.04, 40.16; HRMS (ESI MS) m/z: theor: 413.2202 found: 413.2207 (M^+.^ detected). Analyses were consistent with those previously reported in the literature [17] (See Scheme 4).

### 2.2. Photopolymerization Experiments

In order to study the influence of the [dye-TEA] CTC on the photopolymerization efficiency, the polymerization kinetics of the aqueous monomer PEG-DA in water (50% PEG-DA/50% water) initiated with the [dye-TEA] CTC was monitored by real-time Fourier transform infrared spectroscopy (RT-FTIR, JASCO FTIR 4100, Lisses, France). The photosensitive formulations were dropped between two polypropylene (PP) films with a thickness of 0.1 mm or into molds (between two polypropylene films) with a thickness of 2 mm to reduce O^2^ inhibition. The mixture was irradiated by the LED @ 405 nm lamp, whose light intensity was ~110 mW/cm^2^.

### 2.3. Characterization

Tensile tests of the PEG-polymers initiated with the [dye-TEA] CTC upon irradiation with a LED @ 405 nm were performed by a Dynamometre Instron at a displacement rate of 0.1 mm/min, and the tensile strength at limit was calculated from the subsequent stress/strain curves. The evolution of the dynamic moduli upon photopolymerization was followed on a Haake-Mars Rotational Rheometer (ThermoFisher, Asnières sur Seine, France) at a constant frequency of 10 Hz and at 25 °C. A plate-plate geometry with a gap of 400 micrometers was used. All the experiments were carried out at room temperature [18,19].

### 2.4. Direct Laser Write (DLW) Experiment

Using the dye/TEA CTC as the PIS for PEG-DA, 3D patterns were successfully obtained while using a computer-programmed laser diode (Thorlabs) with a spot size of about 50 μm or a LED projector @ 405 nm as the light radiation source. The resin was spread into a homemade mold with a thickness of about 2 mm, and highly precise 3D patterns were produced through different laser write speeds under air. Finally, the produced 3D patterns were analyzed using a digital optical microscope (DSX-HRSU from Olympus Corporation, Rungis, France) [20].

## 3. Results and Discussion

**Figure 1:**
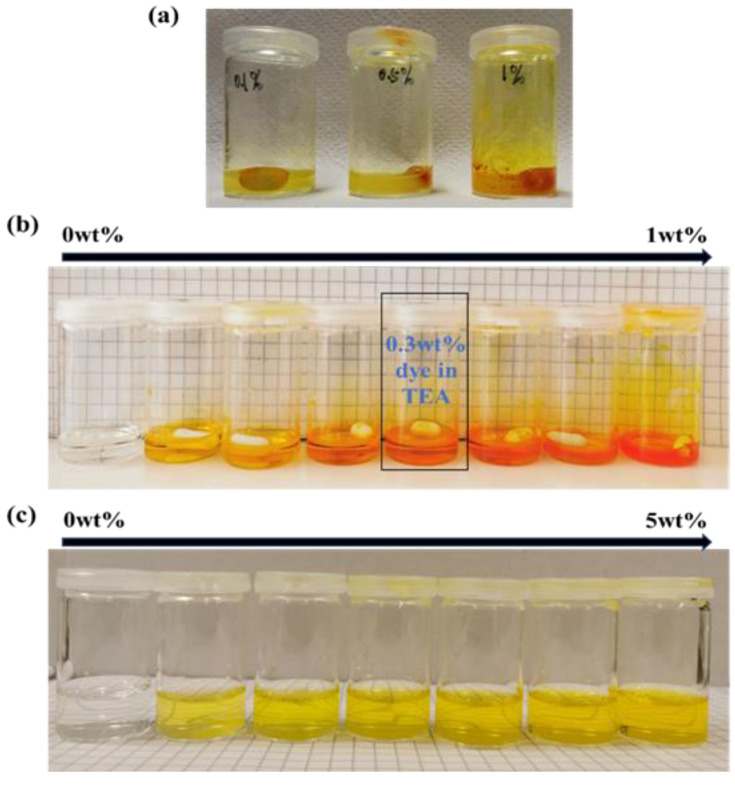
Pictures of (**a**) dye in water with different concentrations from 0.1 wt% to 1 wt%; (**b**) dye in TEA with different concentrations of 0 wt%, 0.05 wt%, 0.1 wt%, 0.2 wt%, 0.3 wt%, 0.4 wt%, 0.5 wt%, 1 wt%; (**c**) [dye-TEA] (0.3 wt% of dye in TEA) in water for different contents: 0 wt%, 0.5 wt%, 1 wt%, 2 wt%, 3 wt%, 4 wt%, 5 wt%.

According to our previous works concerning *mono*-chalcone or *bis*-chalcone-based natural dyes, these compounds are basically soluble in organic solvents (such as acetonitrile) but insoluble or only slightly soluble in water [21,22,23,24,25]. Therefore, during the process of molecular design, we tried first to introduce a section of polyethylene glycol chain into the chalcone structure (as shown in Scheme 1) in order to increase the water solubility. As shown in Figure 1a, when the dye concentration is only 0.1 wt%, although the solution is light yellow and transparent, some dyes still adhered to the surface of the magnetic stirrer. After increasing the concentration to 0.5 wt%, the solution changed from transparent to opaque with the presence of a suspension. The above results indicated that the water solubility of this dye is still very low, which may be due to a too short PEG chain i.e., the central part of the molecule still remains the insoluble chalcone structure.

As the water solubility of chalcone-based dye is limited, and the formation of CTC proved to be a simple and effective method to enhance the solubility in water. In this work, another strategy for preparing water-soluble PIS is proposed, which consists in just mixing the dye exhibiting a poor solubility with a water—oil amphiphilic TEA at the saturation concentration to form the CTC and thus increase the solubility of the dye (Figures S1 and S2). Figure 1b presents the images of the dye into TEA solutions of different concentrations varying from 0 wt% to 1 wt% (more precisely 0 wt%, 0.05 wt%, 0.1 wt%, 0.2 wt%, 0.3 wt%, 0.4 wt%, 0.5 wt% and 1 wt%). The color of the dye/TEA solutions gradually deepened from colorless to yellow, from clear to turbid. Furthermore, we found that the TEA solutions with the dye at the concentration of 0.3 wt% were completely transparent, while the concentration increased to 4 mg/mL (0.4 wt%), although the solution is transparent, small undissolved particles can be observed. Hence, 0.3 wt% might be close to the saturation concentration for the dye in TEA solution and we decided to use this concentration to investigate its water solubility in Figure 1c.

This solution will be noted [dye-TEA]. In order to study the solubility of the dye/TEA combination in water, [dye-TEA] (0.3 wt% dye in TEA) was gradually added into the water at different contents from 0 wt% to 5 wt% (See Figure 1c). Due to the formation of the CTC and hydrogen bonds between the dye and TEA, the [dye-TEA] CTC showed an enhanced solubility in water compared to the dye considered alone (see also in Figures S1 and S2 for the CTC spectroscopic properties). All these [dye-TEA] aqueous solutions were transparent, and their colors changed from colorless to yellow and gradually deepened (See Figure 1c), which revealed that the [dye/TEA] PIS could be soluble in water.

**Figure 2:**
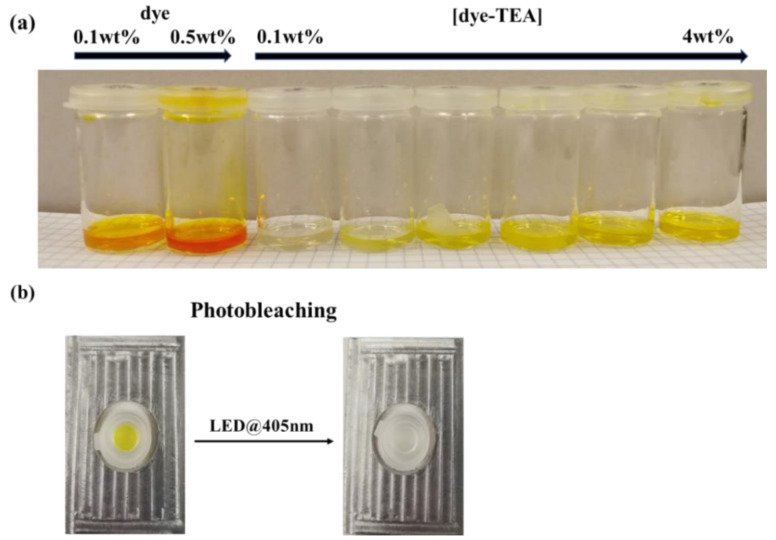
(**a**) Photo-images of dye (0.1 wt%, 0.5 wt%), [0.3wt%dye in Triethanolamine] (0.1 wt%, 0.5 wt%, 1 wt%, 2 wt%, 3 wt%, 4 wt%) in the monomer of PEG-DA (50 wt%)-water (50 wt%); (**b**) Photo-images of photobleaching during the photopolymerization process.

**Figure 3:**
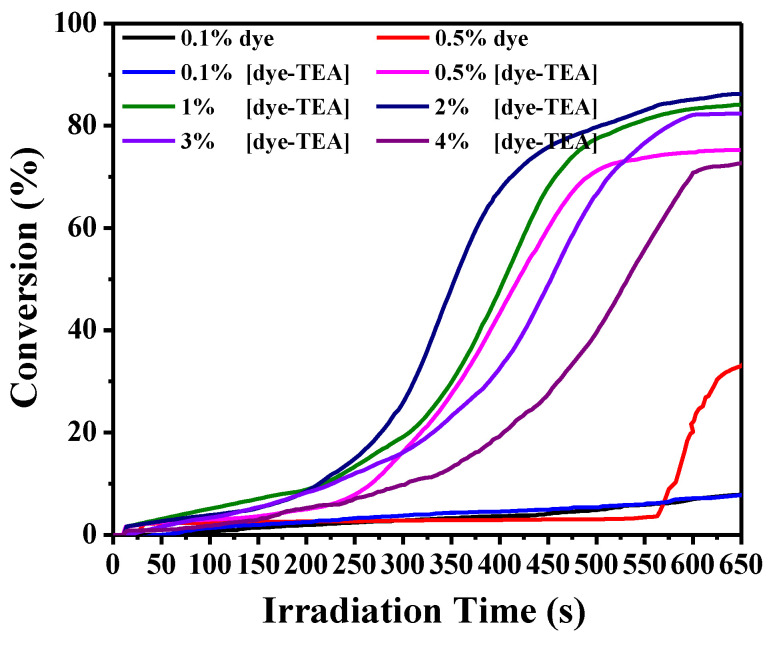
Photopolymerization kinetics (acrylate function conversion vs. time) of PEG-DA (50 wt% PEG-DA/50 wt% water) aqueous monomer initiated by dye (0.1 wt%, 0.5 wt%) and [dye-TEA] (0.1 wt%, 0.5 wt%, 1 wt%, 2 wt%, 3 wt%, 4wt%) in laminate upon irradiation with a LED @ 405 nm with the thickness about 2 mm. The irradiation starts at *t* = 10 s.

In order to study the ability of [dye/TEA] CTC to act as PIS for the free radical polymerization (FRP) of waterborne monomer PEG-DA (50 wt% PEG-DA/50 wt% water), the Real-Time Fourier Transform InfraRed (RT-FTIR) technique was used to monitor the polymerization profiles upon irradiation with a LED @ 405 nm in laminate with the thickness about 0.1 mm and 2 mm (as shown in Figure 2 and Figure 3). The expected photoinitiating mechanism is depicted in Figure S3. Without coinitiator TEA, both poor compatibilities of dye and low final acrylate function conversion of PEG-DA aqueous monomer were observed. In addition, deep curing did not occur at a thickness of about 0.1 mm or 2 mm. However, after adding [dye-TEA] into the PEG-DA aqueous monomer, the dye could be well dispersed in the resin, as shown in Figure 2a.

Moreover, deep curing can now occur with the thickness of about 2 mm with the addition of [dye-TEA] into PEG-DA aqueous monomer except for the concentration of 0.1 wt%, while all of them weren’t fully cured with the thickness of about 0.1 mm. As the concentration of [dye-TEA] increased from 0.1 wt% to 4 wt% (0.1 wt%, 0.5 wt%, 1 wt%, 2 wt%, 3 wt%, 4 wt%), both the acrylate final function conversion and photopolymerization rate of PEG-DA increased remarkably, which indicated the huge effect of the presence of TEA. Interestingly, an optimal concentration for the [dye-TEA] PIS was determined. Indeed, if a final monomer conversion of 85% could be obtained at 2 wt%, the monomer conversion decreased to 83% at 3 wt% dye/TEA PIS. Additionally, the polymerization rate determined at 2 wt% [dye-TEA] PIS was faster than that obtained at 3 wt% [dye-TEA] PIS (the slope of polymerization curves) (See Table 1). This result may be due to the darker color of the photosensitive resin and the faster surface polymerization rate at the concentration of 3 wt%, which inhibited the penetration of light and cause an inner filter effect [26]. More interestingly, the photobleaching phenomenon could also be observed during the process of photopolymerization initiated by the [dye-TEA] PIS and colorless polymers could be advantageously obtained (See Figure 2b).

The mechanical properties of the hydrogels prepared with 2 wt% [dye-TEA] in PEG-DA (50 wt%) aqueous monomer upon irradiation with a LED @ 405 nm were investigated using both a Haake-Mars Rotational Photo-Rheometer and a Dynamometre (Instron), and then compared with the hydrogels prepared with 2 wt% of the commercially available water-soluble photoinitiator (Omnirad 819 DW) with PEG-DA aqueous monomer under ultraviolet light LED @ 365 nm irradiation. Omnirad 819 DW was used here as a benchmark PI for water-based formulations. It consists of a dispersion of 45% *bis*-acylphosphine oxide in water, allowing for stable dispersions.

**Figure 4:**
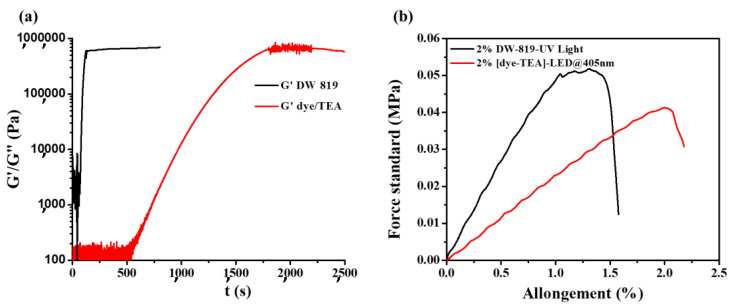
(**a**) The storage (G′) modulus of hydrogels obtained with 2 wt% Omnirad 819 DW under LED @ 365 nm and 2 wt% [dye-TEA] under LED @ 405 nm; (**b**) Tensile test of hydrogels obtained with 2 wt% Omnirad 819 DW under a LED @ 365 nm irradiation and 2 wt% [dye-TEA] upon irradiation with the LED @ 405 nm.

The evolution of the storage (G′) modulus of hydrogels monitored through time-sweep experiments is shown in Figure 4a. Specifically, the storage modulus (G′) of all hydrogels increased with the light irradiation and reached a maximum of about 106 Pa after 20 min for the PEG hydrogels prepared with the [dye-TEA] PIS, while for the PEG hydrogels prepared with Omnirad 819 DW, the maximum storage modulus (G′) reached 106 Pa after 2 min, which was much faster than the proposed [dye-TEA] PIS. Furthermore, as shown in Figure 4b, the tensile strain of the PEG hydrogel prepared with the [dye-TEA] PIS under visible light (LED @ 405 nm) reaches 2% with a tensile strength of about 0.04 MPa. In contrast, the tensile strain of the PEG hydrogel prepared with Omnirad 819 DW under ultraviolet light (LED @ 365 nm) is only about 1.5 wt% with a tensile strength of 0.05 MPa.

**Figure 5:**
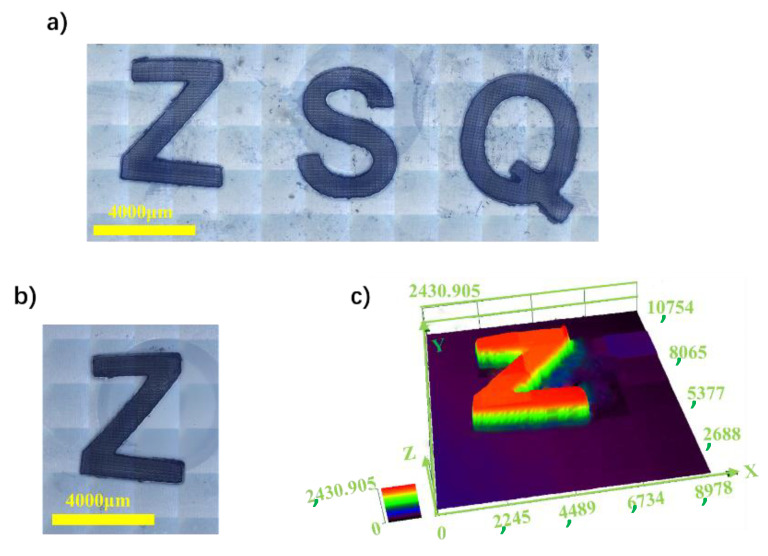
The 3D morphologies of the 3D patterns obtained through the direct laser write experiment initiated with 2wt% [dye-TEA] in PEG-DA aqueous monomer characterized by numerical optical microscope: (**a**) “ZSQ” pattern and (**b**,**c**) zoom on “Z” pattern.

The FRP process of waterborne monomer PEG-DA could also be studied by direct laser write experiments under air using the 2 wt% [dye-TEA] PIS due to its remarkable photosensitivity at 405 nm. As shown in Figure 5, the 3D pattern “Z” exhibiting a remarkable spatial resolution (size of the laser spot) could be successfully fabricated upon irradiation with a LED @ 405 nm, in which the writing process needs a longer time due to its slow polymerization rate. After that, further profilometric observations with a numerical optical microscope were used to characterize the letter patterns. All the results showed that hydrogels with a remarkable spatial control could be prepared with the proposed [dye/TEA] PIS.

## 4. Conclusions

In conclusion, by simple in-situ mixing of the proposed water-slightly soluble PI chalcone-based dye and the oil-water amphiphilic coinitiator TEA, a stable water-soluble complex was obtained through the CTC effect and hydrogel bond. This water-soluble complex [dye-TEA] CTC could be used as an effective PIS to initiate the FRP of the aqueous monomer PEG-DA under visible light irradiation (LED @ 405 nm). Furthermore, the obtained hydrogel demonstrated good mechanical properties. In addition, 3D patterns with spatial resolution were successfully obtained through direct laser write experiments by using this proposed [dye-TEA] based-PIS. Therefore, the proposed [dye-TEA] CTC can be used in the field of 3D printing and hydrogel preparation under visible light.

## Figures and Tables

**Table 1:** Summary of the final function conversions of waterborne monomers (PEG-DA) under LED @ 405 nm initiated by dye (0.1 wt%, 0.5 wt%) and [dye-TEA] (0.1 wt%, 0.5 wt%, 1 wt%, 2 wt%, 3 wt%, 4 wt%) in laminate upon LED @ 405 nm with a thickness of about 2 mm.

Final Acrylate Function Conversions of PEG-DA Aqueous Monomers	
0.1% dye	8%
0.5% dye	33%
0.1% [dye-TEA]	8%
0.5% [dye-TEA]	75%
1% [dye-TEA]	84%
2% [dye-TEA]	86%
3% [dye-TEA]	82%
4% [dye-TEA]	72%

## Data Availability

No samples available.

## Supplementary Materials

The following are available online at https://www.mdpi.com/article/10.3390/polym13183195/s1, Figure S1. The normalized absorption and emission spectrums of [dye-TEA] CTC in water. Figure S2. The normalized absorption of dye and [dye-TEA] CTC in acetonitrile. Figure S3. The involved mechanism of [dye-TEA] CTC.
