# Supplementary material for: Water-Soluble Visible Light Sensitive Photoinitiating System Based on Charge Transfer Complexes for the 3D Printing of Hydrogels
Source: Polymers (Basel). 2021 Sep 21;13(18):3195. doi: 10.3390/polym13183195 (PMC8470713; doi:10.3390/polym13183195)
Supplement: Supplementary file 1 [file polymers-13-03195-s001.zip › polymers-1356064-supplementary.pdf]

polymers

MDPI

Supplementary Material

Water-Soluble Visible Light Sensitive Photoinitiating System Based on Charge Transfer Complexes for the 3D Printing of Hydrogels

Hong Chen [1,2], Mehdi Vahdati [1,2], Pu Xiao [3,*], Frédéric Dumur [4,*] and Jacques Lalevée [1,2,*]

1 Université de Haute-Alsace, CNRS, IS2M UMR 7361, F-68100 Mulhouse, France; hong.chen@uha.fr (H.C.); mehdi.vahdati@uha.fr (M.V.)
2 Université de Strasbourg, France
3 Research School of Chemistry, Australian National University, Canberra ACT 2601, Australia
4 Aix Marseille Univ, CNRS, ICR UMR 7273, F-13397 Marseille, France
* Correspondance: pu.xiao@anu.edu.au (P.X.); frederic.dumur@univ-amu.fr (F.D.); jacques.lalevee@uha.fr (J.L.)

Citation: Chen, H.; Vahdati, M.; Xiao, P.; Dumur, F.; Lalevée, J. Water-Soluble Visible Light Sensitive Photoinitiating System Based on Charge Transfer Complexes for the 3D printing of Hydrogels. *Polymers* **2021**, *13*, 3195. https://doi.org/10.3390/polym13183195

Academic Editor: Chin-San Wu

Received: 10 August 2021
Accepted: 18 September 2021
Published: 21 September 2021

Publisher's Note: MDPI stays neutral with regard to jurisdictional claims in published maps and institutional affiliations.

Copyright: © 2021 by the authors. Licensee MDPI, Basel, Switzerland. This article is an open access article distributed under the terms and conditions of the Creative Commons Attribution (CC BY) license (http://creativecommons.org/licenses/by/4.0/).

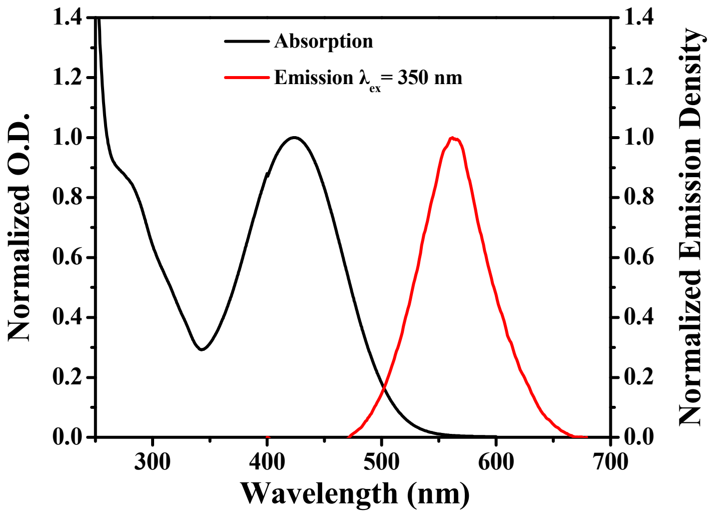

Figure S1. The normalized absorption and emission spectrums of [dye-TEA] CTC in water.

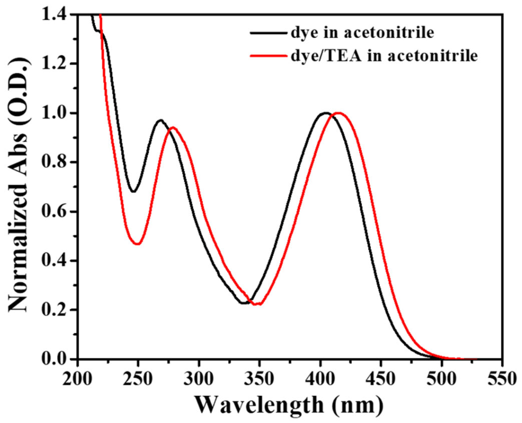

Figure S2. The normalized absorption of dye and [dye-TEA] CTC in acetonitrile.

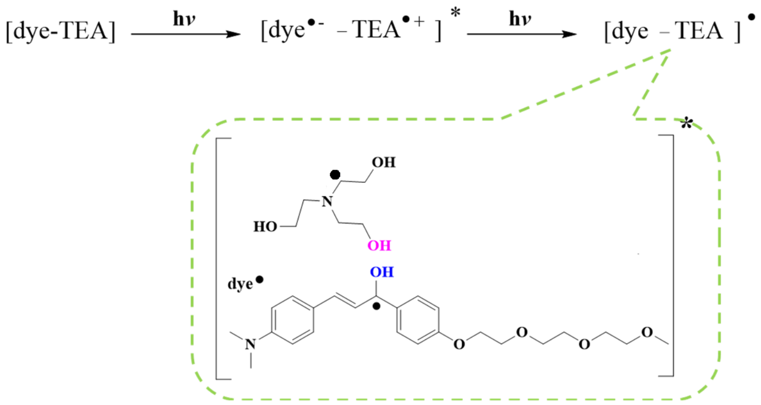

Figure S3. The involved mechanism of [dye-TEA] CTC.
